# Flaxseed Influences Basic Health Parameters, Fatty Acid Content Cell Membrane Integrity, Cell Proliferation and Angiogenesis in Fattening Pigs

**DOI:** 10.3390/life16081380

**Published:** 2026-08-21

**Authors:** Zuzana Andrejčáková, Radoslava Vlčková, Zdenka Hertelyová, Katarzyna Kozioł, Kristína Šmajda Rodáková, Drahomíra Sopková

**Affiliations:** 1 Department of Biology and Physiology, University of Veterinary Medicine and Pharmacy in Košice, Komenského 73, 041 81 Košice, Slovakia; zuzana.andrejcakova@uvlf.sk (Z.A.); kristina.rodakova@student.uvlf.sk (K.Š.R.); drahomira.sopkova@uvlf.sk (D.S.); 2 Center for Clinical and Preclinical Research Medipark, Faculty of Medicine, Pavol Jozef Šafárik University in Košice, Šrobárova 2, 041 80 Košice, Slovakia; zdenka.hertelyova@upjs.sk; 3 Institute of Biotechnology, College of Natural Sciences, University of Rzeszow, Pigonia 1, 35-310 Rzeszow, Poland

**Keywords:** flaxseed, fattening pigs, lactate dehydrogenase, polyunsaturated fatty acids, CD31, PCNA

## Abstract

This study focused on dietary supplementation with flaxseed in fattening pigs to observe health benefits and important biological findings, including cell membrane integrity, proliferation, and angiogenesis. Animals received flaxseed for 6 or 3 weeks prior to slaughter. Pigs were divided into a control group (standard diet) and experimental groups F6 (6 weeks) and F3 (3 weeks), which received flaxseed in the feed. Spectroscopy followed by an electrophoretic method was used to study the levels of basic biochemical parameters, including LDH. Subsequently, gas chromatography revealed fatty acid concentrations in the pigs’ livers, and finally, immunohistochemical analysis was used to detect CD31 and PCNA antigens in the liver and jejunum. The highest weight gain was measured in the F6 group. After slaughter, blood samples revealed a decreased glucose level in the F6 group, maintained levels of basic biochemical parameters, and decreased concentrations of LDH in blood, heart, liver, and skeletal muscle extracts, thus improving cell membrane integrity. A long-term supplementation period led to better utilization of essential fatty acids, an improved n-6:n-3 ratio, and other atherogenic indices related to cardiovascular health. Flaxseed also affected the expression of CD31 and PCNA proteins in the liver and jejunum, mainly in the F6 group.

## Simple Summary

Flaxseed is a natural source of bioactive compounds, particularly omega-3 fatty acids, and may provide health benefits when included in animal diets. This study investigated the effects of flaxseed supplementation in fattening pigs for 3 or 6 weeks. Animals were assigned to a control group or groups receiving flaxseed for 3 weeks (F3) or 6 weeks (F6). Growth performance, biochemical parameters, lactate dehydrogenase (LDH), liver fatty acid composition, and PCNA and CD31 expression in the liver and jejunum were evaluated. Six-week supplementation was associated with greater weight gain, lower blood glucose levels, and reduced LDH concentrations in blood and tissues, indicating improved cellular membrane stability. It also increased the levels of selected fatty acids, including ALA, EPA, DHA, and AA. Changes in PCNA and CD31 expression, particularly in the F6 group, suggest that flaxseed influences cellular proliferation and vascularization-related processes. Overall, six-week supplementation may support metabolic status and tissue health in fattening pigs.

## 1. Introduction

Flaxseed (*Linum usitatissimum*), an ancient crop, has attracted increasing scientific and nutritional interest due to its rich composition, including n–3 fatty acids (FA), proteins, lignans, and fiber. Duarte et al. [1] highlighted that n–3 polyunsaturated fatty acids (PUFAs), especially alpha linolenic acid (ALA, 18:3n-3), improve cardiovascular health, cancer prevention, and glycemic regulation and promote gastrointestinal well-being. Among various constituents, essential linoleic acid (LA, 18:2n 6) is a precursor of n–6 PUFAs and pro-inflammatory eicosanoids. N-6 PUFAs are very important for maintaining the physiological functions of the brain, cardiovascular system, and normal growth and development. Lack of n-6 FA in the diet causes slow wound healing and dry hair with subsequent hair loss. Excessive content of n-6 PUFAs in the body has proatherogenic, proinflammatory, and prothrombotic effects [2,3,4].

Flaxseed is considered one of the richest dietary sources of plant lignans, particularly secoisolariciresinol diglucoside (SDG), which is converted by intestinal bacteria into mammalian lignans (enterodiol and enterolactone), with antioxidant properties and, due to their similar structure to estrogen, estrogen-balancing effects, potentially reducing the risk of breast, endometrial, and prostate cancers [5,6].

Flaxseed and its derivatives significantly enhance cell membrane integrity and fluidity through the high concentration of ALA. Alpha linolenic acid and its metabolites (eicosapentaenoic acid, EPA, and docosahexaenoic acid, DHA) are incorporated into the cell membrane phospholipid bilayer, modulating its fluidity and physicochemical properties [7,8]. Moreover, flaxseed and its components have the ability to improve cell membrane integrity after toxic exposure [9], as measured by lactate dehydrogenase (LDH) activity. Studies investigating inflammatory conditions have demonstrated that LDH enzyme activity is associated with inflammatory processes and tissue disruption [10].

Pork represents an important component of the human diet, providing high-quality protein with a favorable amino acid profile, essential fatty acids (FAs), B-group vitamins, and essential trace elements, including zinc, selenium, and iron [11]. In monogastric animals the dietary fatty acid profile can be effectively reflected in muscle tissue, providing an opportunity to increase the deposition of beneficial n-3 FAs through dietary supplementation. In pigs, dietary inclusion of whole flaxseed has been shown to increase n-3 FA concentrations in various tissues without adversely affecting growth performance or meat quality during storage [12]. At a 27.8% concentration, flaxseed served as a new protein feed raw material [13,14], while at a 2.5% concentration, it significantly increased n-3 PUFA levels in meat and backfat [15]. The levels of flaxseed supplementation, up to 5%, have been investigated by Matthews et al. [12], Kouba et al. [16] and other authors. Juárez et al. [17] reported that higher levels of flaxseed inclusion, up to 20%, did not adversely affect growth performance in fattening pigs.

We hypothesized that dietary supplementation with 10% flaxseed for 3 or 6 weeks would beneficially affect the metabolic health of fattening pigs by improving serum biochemical parameters and liver fatty acid composition. We also studied whether prolonged flaxseed supplementation (6 weeks) would exert greater beneficial effects than short-term supplementation (3 weeks).

Additionally, we hypothesized that flaxseed would enhance cell membrane integrity, as evidenced by lower LDH activity, and promote tissue regeneration through modulation of cell proliferation and angiogenesis, reflected by changes in the expression of proliferating cell nuclear antigen (PCNA) and platelet endothelial cell adhesion molecule (CD31).

## 2. Materials and Methods

### 2.1. Animal Housing and Diets

A total of 18 female Landrace pigs (20 weeks of age) were included in the experiment. The study lasted either 3 or 6 weeks until slaughter at Slaughter Station Inc. (Vajanského Street 789, Spišské Vlachy, Slovakia). No clinical signs of disease were observed in any of the animals during the experimental period.

Pigs were housed in pens with two animals per pen and had ad libitum access to water via nipple drinkers. The average ambient temperature and relative humidity were maintained at 18 °C and 60%, respectively. The health status of the animals and fecal consistency were monitored daily throughout the experimental period. During the experiment, parasitological and bacteriological examinations of fecal samples and nasal swabs were performed, with no positive findings detected in any of the animals.

Body weight measurements were recorded three times during the flaxseed supplementation period: at the beginning of the experiment (Day 0), in the middle (Day 11 or Day 21), and at the end of the experiment (Day 21 or Day 42). Then, average daily gain (ADG) was calculated. Blood samples were collected at the same time points.

Animals were randomly assigned to three groups (n = 6 per group). The control group (C) received a commercial complete feed mixture for fattening pigs (maize—13%; alfalfa—36.5%; wheat—32%; soybean meal—8.5%; wheat bran—7%; bicalcium phosphate—1.29%; monocalcium phosphate—0.78%; sodium chloride—0.45%; mineral premix 0.48%). The first experimental group (F6) received the same feed supplemented with 10% flaxseed for 6 weeks, whereas the second experimental group (F3) received the supplemented diet for 3 weeks. Flaxseed of the Libra variety containing 57% α-linolenic acid (ALA) was used. Both the flaxseed and the commercial feed mixture were obtained from Dom krmív (Spišské Vlachy, Slovakia). The daily feed ration was 3 kg per animal. However, individual feed intake was not recorded; therefore, the feed conversion ratio (F/G) was not calculated. The nutritional composition of all types of feed is stated in Table 1.

During the experiment, the following blood biochemical parameters were determined: glucose, aspartate aminotransferase (AST), alanine aminotransferase (ALT), alkaline phosphatase (ALP), gamma-glutamyl transferase (GGT), creatine kinase (CK), total protein (TP), total lactate dehydrogenase (TLDH), and lactate dehydrogenase isoenzymes (LDH-1 to LDH-5).

On the day of slaughter (Day 21 or Day 42), samples of the heart, liver, and skeletal muscle (musculus gluteobiceps) were collected for determination of TLDH activity and LDH isoenzyme profiles. Tissue samples (2 g) were cut into small pieces and homogenized in 20 volumes of cold 0.05 M Tris-HCl buffer (pH 7.3) according to Heinová et al. [18]. Protein concentration was determined according to Bradford [19]. Total LDH activity and the activities of LDH isoenzymes (LDH-1 to LDH-5) were analyzed by spectrophotometric and electrophoretic methods and expressed as international units per gram of protein (IU/g protein). Liver samples were additionally subjected to fatty acid analysis using gas chromatography. Moreover, 1 cubic centimeter of liver tissue was cut for immunohistochemical analysis for the presence of anti-CD31 and anti-PCNA antibodies.

### 2.2. Spectroscopy Analysis

Blood serum concentrations of TLDH, ALT, CK, AST (λ = 340 nm), ALP, GGT (λ = 405 nm), glucose (λ = 500 nm), and TP (λ = 546 nm) were determined using commercial diagnostic kits (Randox, Crumlin, UK). Analyses were performed using an Alizé automatic biochemical analyzer (Lisabio, Morangis, France).

### 2.3. Electrophoretic Analysis

For electrophoretic analysis (using a Hydrasys device, Sebia, Lisses, France), samples of blood serum and tissue homogenates prepared from the heart muscle, liver, or skeletal muscle of fattening pigs were used. The samples were separated using commercial Hydragel 15 ISO-LDH electrophoretic kits according to our previous research [9]. Densitometric curves of the separations were created by means of an Epson Perfection V700 Photo densitometer (λ 570 nm), and the resulting profiles were evaluated using Phoresis software (Version 5.50, 2009, Sebia, Lisses, France).

### 2.4. Gas Chromatography

Extraction of fatty acids (FA) from liver tissue homogenates and subsequent gas chromatographic analysis were performed according to Folch et al. [20]. Extracted lipids were transesterified to fatty acid methyl esters (FAMEs) using sodium methanolate. The FAME profile was determined using a gas chromatograph (Shimadzu GC-17, Shimadzu, Kyoto, Japan) equipped with a flame ionization detector. Results were expressed as mol%.

### 2.5. Histological and Immunohistochemical Analysis

Liver (caudate lobe) and jejunum (middle part) samples from each group of pigs (n = 6/group) were fixed in 4% paraformaldehyde, dehydrated through graded ethanol, and embedded in paraffin. Sections (5 μm) were stained with hematoxylin and eosin (H&E) for histological evaluation.

For immunohistochemistry, 4 μm sections were prepared, rehydrated, and subjected to heat-induced epitope retrieval in citrate buffer (pH 6.0). For immunohistochemistry, 4 μm sections were prepared, rehydrated, and subjected to heat-induced epitope retrieval in citrate buffer (pH 6.0). Endogenous peroxidase activity and non-specific binding were blocked with 1% H_2_O_2_ and 1% bovine serum albumin (BSA), respectively. Sections were incubated overnight at 4 °C with primary antibodies against proliferating cell nuclear antigen (PCNA; Santa Cruz Biotechnology, Dallas, TX, USA, Cat. No. sc-56; 1:250) and platelet endothelial cell adhesion molecule-1 (CD31; Abcam, Cambridge, UK, Cat. No. ab28364; 1:50), followed by incubation with Dako REAL EnVision/HRP secondary antibodies for 2 h. Immunoreactivity was visualized using 3,3′-diaminobenzidine (DAB), and sections were counterstained with hematoxylin. Images were acquired using an Optica B-290TB light microscope (Optika Microscopes Italy, Ponteranica, Italy) and analyzed with ImageJ software (v. 1.53t). Relative optical density (ROD) was calculated according to Smolen [21]. Detailed immunohistochemical procedures have been described previously [9,22].

### 2.6. Statistical Analyses

Statistical analyses were performed using GraphPad Prism 8.3 (GraphPad Software, San Diego, CA, USA). Data are presented as the mean ± standard error of the mean (SEM). Differences among groups were evaluated using one-way analysis of variance (ANOVA) followed by Tukey’s post hoc test. Statistical significance was established at *p* < 0.05.

## 3. Results

### 3.1. Weight Changes in Fattening Pigs

**Figure 1:**
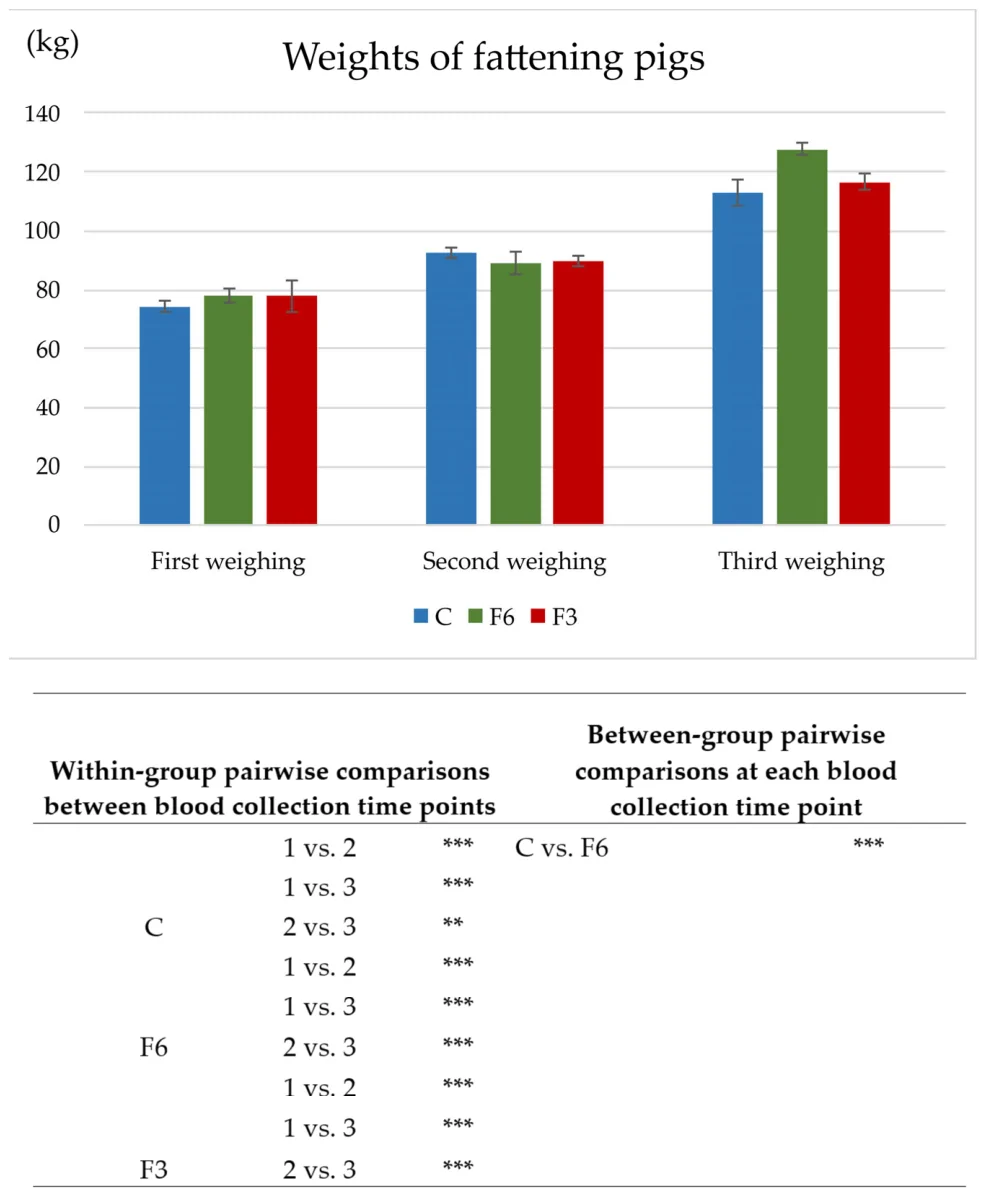
Weight changes in fattening pigs during the flaxseed supplementation period. C, control group; F6, flaxseed fed for 6 weeks; F3, flaxseed fed for 3 weeks; first weighing or Day 0 of the experiment; second weighing (Day 11 or 21); third weighing or the end of the experiment (Day 21 or 42); values are mean ± SEM, where ** = *p* < 0.001, *** = *p* < 0.001.

Changes in weight (Figure 1) of fattening pigs were observed during the supplemental feeding period (10% flaxseed), which lasted 3 and 6 weeks. The most significant weight gain was observed in pigs fed flaxseed for 6 weeks at the end of the experiment (*p* < 0.05) compared to the control. Each group showed higher weight gain (*p* < 0.001) compared to the first measurement. Average daily gain (ADG) values were also calculated for each group. The ADG value for the C group was 0.925 ± 0.326 kg/day; in the F6 group the value reached 1.175 ± 0.237 kg/day, and in the F3 group the ADG value was 2.008 ± 0.684 kg/day. Significant elevation was noticed in the F3 group compared to the control or the F6 group (both *p* < 0.05).

### 3.2. Changes in Basic Blood Parameters During the Flaxseed Feeding Period in Pigs

Table 2 and Table 3 show changes in selected blood parameters in fattening pigs. Glucose levels were not affected by flaxseed feeding during the experiment, except for the F6 group, which received flaxseed at a 10% concentration (*p* < 0.05), where glucose levels markedly decreased compared to the control. The activity of AST was not affected during the entire experiment; only the F6 group showed a slight decrease in the middle of the experiment (*p* < 0.05; 2nd blood collection) compared to the control. The concentration of ALT decreased in the control (*p* < 0.001) and the F6 (*p* < 0.05) groups during the second and third blood collections. Similarly to AST, a slight decrease in ALT values in the middle of the experiment (*p* < 0.05; 2nd blood collection) compared to the control was observed. The ALP activity varied during the three examinations. In the control group, its activity first decreased (*p* < 0.001), and, at the end of the experiment, the values slightly increased (*p* < 0.001). In the F6 group, ALP activity decreased (*p* < 0.05) at the 2nd blood sampling. No significant changes were recorded between the groups at the end of the experiment. The activity of GGT in the control group decreased during the second and third blood samplings (*p* < 0.001). Significantly higher values of GGT were measured in both experimental groups compared to the control (*p* < 0.05) at the end of the experiment. At the second blood collection, creatine kinase was markedly elevated only in the F3 group compared to the first blood sampling (*p* < 0.001). Significantly higher CK activity was observed in the F6 group compared to the F3 group (*p* < 0.05) at the end of the experiment. During the entire experimental feeding period, total protein content in blood significantly increased, mainly in the F6 group (*p* < 0.001). At the end of the experiment, we observed higher values of TP in the F6 group compared to the control (*p* < 0.05).

### 3.3. Lactate Dehydrogenase Activity in Blood Serum of Fattening Pigs

Serum activity of total LDH and its isoenzymes was significantly affected during the experiment (Table 4 and Table 5). During selected blood collections, TLDH values in the control (*p* < 0.05) and F3 (*p* < 0.01) groups slightly increased. The most significant decrease in its activity was measured in the F6 group compared to the control (*p* < 0.05) at the end of the experiment. The LDH-1 concentration generally increased during the experiment in the control group (*p* < 0.05) and the F6 (*p* < 0.001) groups compared to the beginning of the experiment.

Significantly lower concentrations of LDH-1 in the F6 group compared to the control (*p* < 0.001) were measured at the second blood sampling. Isoform LDH-2 varied during the experiment. Significantly lower activity was measured in the control and F6 groups (*p* < 0.05) compared to the first examination. The F6 group showed markedly lower values of LDH-2 (*p* < 0.001) compared to the control at the end of the experiment. The LDH-3 concentration had a rising trend in the control (*p* < 0.01) and F3 group (*p* > 0.05), but the F6 group (*p* < 0.01) showed a decrease in levels. At the end of the experiment, the LDH-3 concentration decreased in the F6 group compared to the control (*p* < 0.01). The dynamics of LDH-4 concentration showed a decline in the F6 group (*p* < 0.01) and an increase in the F3 group (*p* < 0.001). Only one significant increase in LDH-4 in the F6 group (*p* < 0.001) was measured at the beginning of the experiment. A similar trend was measured in LDH-5 isoenzyme activity, where the F6 group (*p* < 0.01) showed decreased concentrations, while the control and F3 groups (both *p* < 0.01) showed increased concentrations. At the end of the experiment, markedly higher concentrations of LDH-5 compared to the control and F3 groups were recorded (both *p* < 0.001).

### 3.4. Lactate Dehydrogenase Activity in Organs of Fattening Pigs

**Figure 2:**
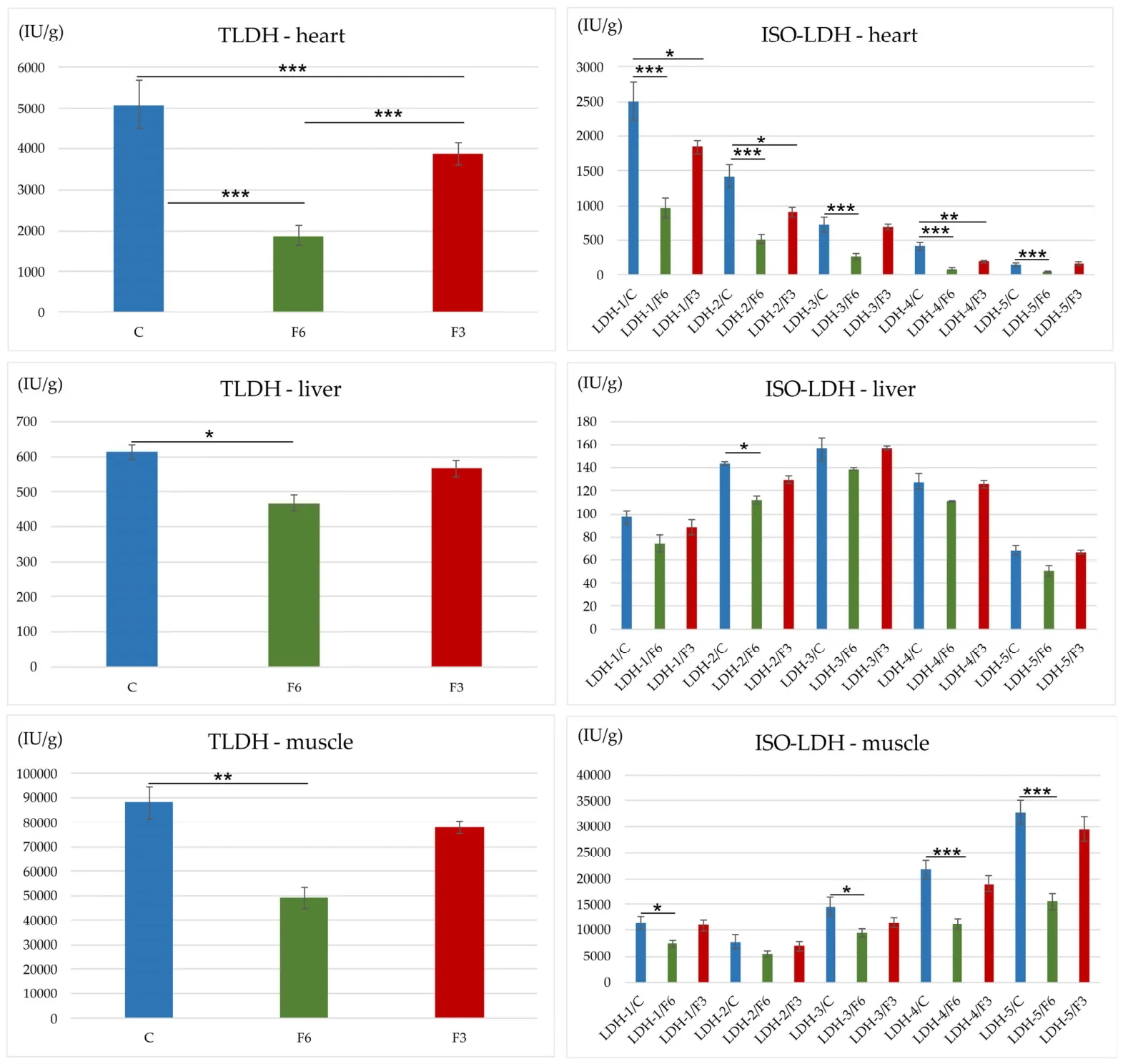
Activity of total lactate dehydrogenase and its isoforms in the heart, liver, and skeletal muscle of fattening pigs. C, control group; F6, flaxseed fed for 6 weeks; F3, flaxseed fed for 3 weeks; TLDH, total lactate dehydrogenase; LDH-1 to LDH-5, isoforms of LDH; values are mean ± SEM, where the asterisks indicate *, *p* < 0.05; **, *p* < 0.01; ***, *p* < 0.001 compared to the control.

The results of the activity of TLDH and its isoforms in organs are shown in Figure 2. Heart muscle showed a significant decrease in TLDH activity in both experimental groups compared to the control (both *p* < 0.001). Similar results were measured in all isoforms. The most visible decline in LDH concentrations (LDH-1 to LDH-5 isoenzymes) was measured in the F6 group, which received 10% flaxseed for 6 weeks (*p* < 0.001). In the F3 group, we observed decreased activity of LDH-1, LDH-2 (both *p* < 0.05), and LDH-4 (*p* < 0.01) compared to the control group. TLDH activity of liver homogenates decreased only in the F6 group (*p* < 0.05). Similarly to the liver, TLDH activity of skeletal muscle decreased only in the F6 group (*p* < 0.05) compared to the control. Reduced activity of LDH-1 and LDH-3 (both *p* < 0.05) and LDH-4 and LDH-5 (both *p* < 0.001) was observed.

### 3.5. Liver Fatty Acid Profile in Fattening Pigs

The liver fatty acid profile was markedly influenced by the addition of flaxseed to the feed of fattening pigs (Table 6 and Table 7). The highest concentration of lauric acid (12:0) was measured in the F3 group compared to the control (*p* < 0.05). We observed the highest value of stearic acid (18:0) in the F6 group (*p* < 0.05) and the lowest value in the F3 group (*p* < 0.01) compared to the control group. The concentration of linoleic acid (18:2n-6; LA) was higher in the control group compared to both experimental groups (both *p* < 0.001). On the other hand, the level of gamma-linolenic acid (18:3n 6; GLA) was highest in the F3 group compared to the control (*p* < 0.001) but also elevated in the F6 group. At the end of the experiment, we measured the highest concentration of alpha-linolenic acid (18:3n-3; ALA) in the F6 group compared to the control or the F3 group (both *p* < 0.001). A similar trend was noticed in dihomo-gamma-linoleic acid (20:3n-6; DGLA), where we recorded the highest concentration in the F6 group compared to the control (*p* < 0.001) and the F3 group (*p* < 0.05). An elevated level of arachidonic acid (20:4n-6; AA) was measured in the control group compared to the F6 and F3 groups (both *p* < 0.01). In the group that received flaxseed for a longer period, the level of timnodonic acid (20:5n-3; EPA) was significantly higher compared to the control and F3 groups (both *p* < 0.001). The concentration of osbond acid n-6 (22:5n-6; DPA-6) was elevated in the group fed flaxseed for a shorter period compared to the control and F6 groups. The n-3 fatty acid clupanodonic acid (22:5n-3; DPA-3) revealed the highest value in the F6 group compared to the control (*p* < 0.001) and the F3 group (*p* < 0.01). The same results were recorded for cervonic acid (22:6n-3; DHA), with similar statistical significance (*p* < 0.001). We measured the highest sum of n-3 fatty acids in the group of animals that received flaxseed for a longer period compared to the control and F3 groups (both *p* < 0.001). Logically, the highest sum of n-6 fatty acids was recorded in the control group compared to both experimental groups (both *p* < 0.001). The same results were observed in calculations of the n-6:n-3 ratio (both *p* < 0.001). The EPA to AA ratio was highest in the F6 group compared to the control and F3 groups (both *p* < 0.001). The same trend was recorded for the calculation of the n-3 index (both *p* < 0.001).

### 3.6. Immunohistochemical Analysis of the Liver and Jejunum

**Figure 3:**
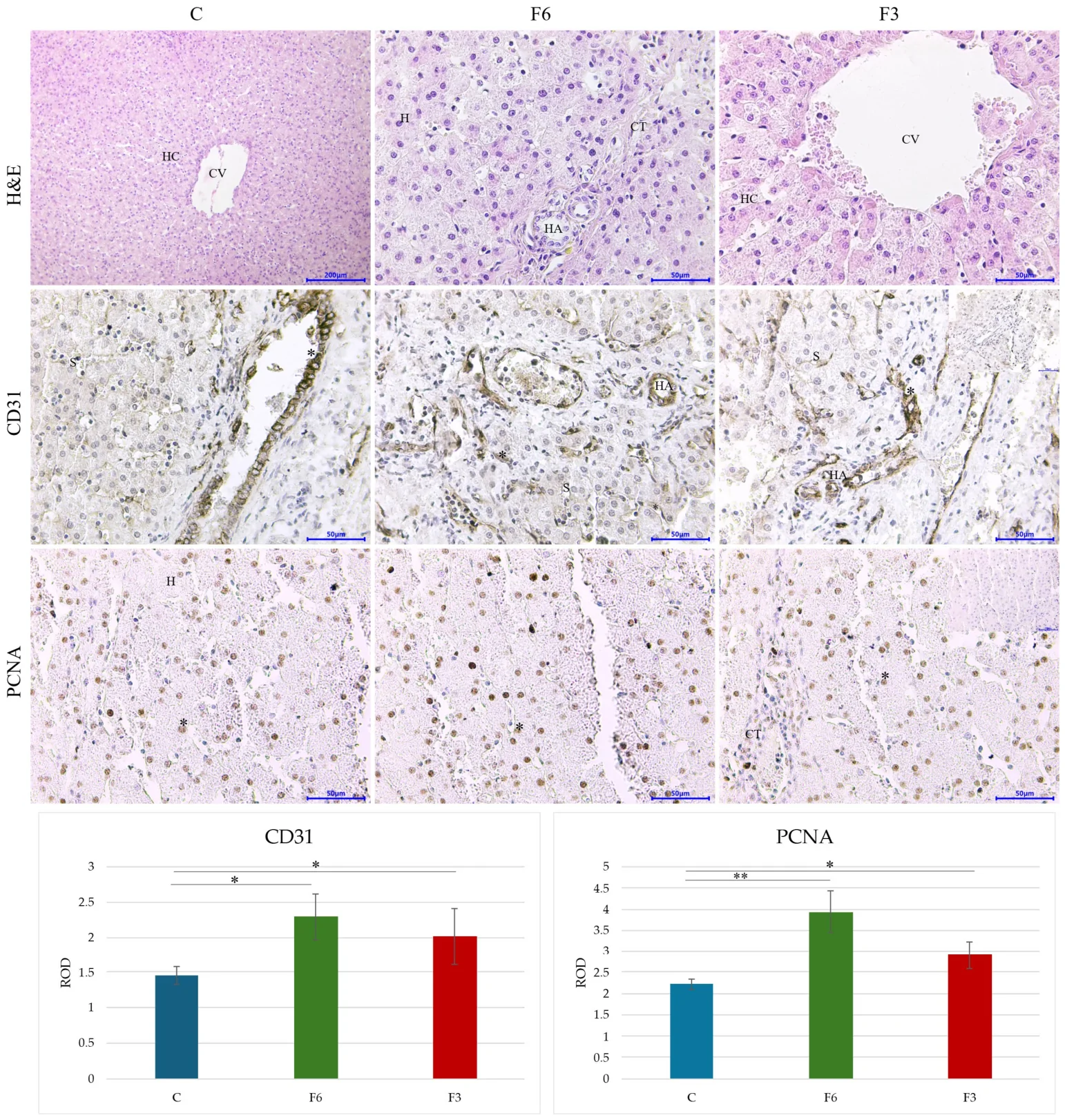
Immunohistochemical analysis of CD31 and PCNA in the liver of fattening pigs. C, control group; F6, flaxseed fed for 6 weeks; F3, flaxseed fed for 3 weeks; CV, central vein; HC, hepatic cords; H, hepatocytes; CT, connective tissue of lobular boundaries; HA, hepatic arteriole; The asterisk (inside of photo) indicates a positive reaction; magnification, 400x, except for the first image (100×); quantification of the intensity of the immunohistochemical reaction and expression of CD31 and PCNA in the liver expressed as the relative optical density (ROD) of the section; values are mean ± SEM, where the asterisks indicate * = *p* < 0.05, ** = *p* < 0.01.

**Figure 4:**
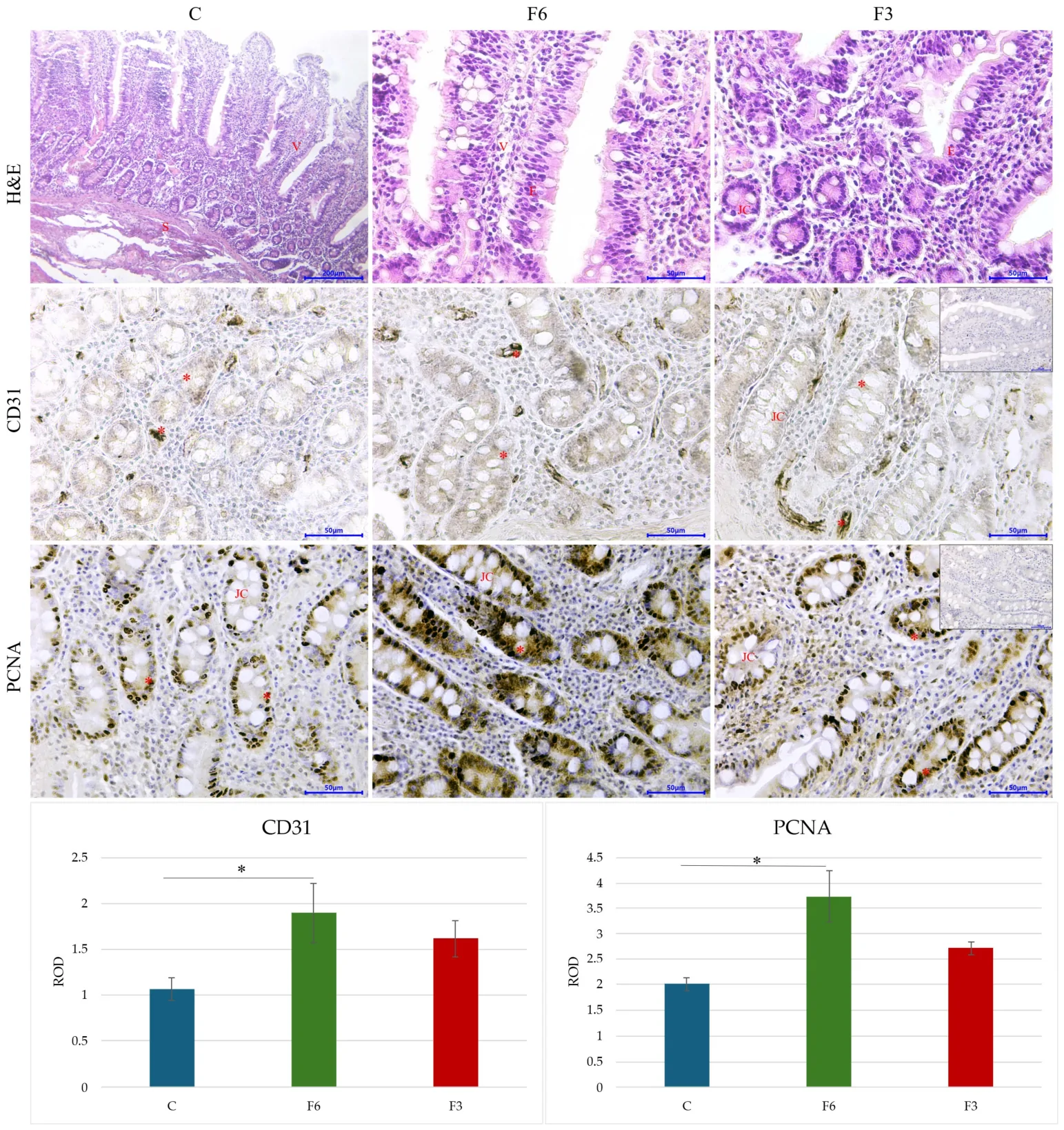
Immunohistochemical analysis of CD31 and PCNA in the jejunum of fattening pigs. C, control group; F6, flaxseed fed for 6 weeks; F3, flaxseed fed for 3 weeks; V, villus; E, enterocyte; JC, jejunal crypt; S, submucosa; The asterisk (inside of photo) indicates a positive reaction; magnification, 400×, except for the first image (100×); quantification of the intensity of the immunohistochemical reaction and expression of CD31 and PCNA in the liver expressed as the relative optical density (ROD) of the section; values are mean ± SEM, where the asterisk indicates * = *p* < 0.05.

Prior to immunohistochemical analysis, the basic histology of the liver and jejunum was assessed (Figure 3 and Figure 4). No pathological changes in the selected organs were recorded. To study the effect of flaxseed on liver function and growth, PCNA and CD31 antigens were detected (Figure 3). In the liver of the pigs, elevated atherogenesis was recorded. The positive brown reaction and expression of CD31 were situated in the liver sinusoids and the lining veins and arterioles. Liver tissue from experimental pigs expressed higher ROD values of CD31 (both *p* < 0.05) compared to control animals. Proliferation of the liver was seen mainly in the nuclei of hepatocytes (brown reaction) and was significantly higher in the F6 group compared to the control (*p* < 0.05). In addition, the F3 group revealed a slight elevation compared to the control (*p* < 0.05). Similar results for those parameters in the jejunum of pigs were observed (Figure 4). The brown reaction of the CD31-positive staining was situated partially in the cytoplasm of enterocytes, and a stronger reaction was seen in arterioles of the submucosa of the jejunum. The positive reaction of the PCNA was situated in the jejunal crypts. The highest ROD values of CD31 and PCNA revealed the same tendency toward elevation compared to the liver. Significant elevation of the ROD values (both antigens) was measured only in the F6 group compared to the control (*p* < 0.05).

## 4. Discussion

Pigs represent a valuable animal model owing to their anatomical and physiological similarities to humans [23,24]; therefore, some findings could be translated to humans and their health status. Moreover, pork is currently one of the most consumed meats worldwide.

This study revealed the highest weight gain after 6 weeks of flaxseed supplementation. Similar results were observed in our previous study [25] and in the study by Soni Guillermo et al. [26], who reported that a 2% concentration of flaxseed in the pig diet for 8 weeks is sufficient to improve meat quality and the resulting fatty acid composition. Many studies have suggested that such a high amount of flaxseed has a negative effect on health status and meat quality and may require more careful management and potentially additional antioxidants to prevent adverse effects such as increased lipid peroxidation [27]. Juárez et al. [28] conducted an experiment with grower-finisher pigs that received 5%, 10%, or 15% flaxseed in the feed for 4, 8, or 12 weeks, and they summarized that a longer feeding period (more than 8 weeks) led to decreased weight gain. This effect could be caused by the high content of water-soluble fiber in flaxseed, which is associated with high water-holding capacity and swelling in the digestive tract, and subsequently reduced nutrient absorption [29]. Soluble fiber in the diet may act as a prebiotic [30,31], and lignans and selected PUFAs from flaxseed downregulate pro-inflammatory cytokines and reduce inflammation. This effect is beneficial because, during inflammation in pigs, the body synthesizes inflammatory mediators instead of using nutrients as building materials and energy sources for growth [32]. Average daily gain (ADG) values revealed elevation mainly in the F3, but also in the F6 group compared to the control. This finding is not in consensus with Soni-Guillermo [26], who claimed that 4% and more of flaxseed in the pig diet caused no effect on ADG and other carcass characteristics. Increased ADG values were recorded only in smaller pigs under 75 kg. Similar results were recorded in the study of Juárez et al. [28] with 10% flaxseed in the feed. In comparison to our study, differences could be caused by different supplementation periods (3 and 6 weeks) and a higher final body weight of pigs on the day of slaughter.

The liver is the largest internal organ, and it has essential functions, including nutrient and drug metabolism, detoxification, and other metabolic processes [33]. In our study, we measured glucose levels and basic biochemical enzymes, including LDH, during three sampling points at the beginning (Day 0), in the middle (Day 11 or 21), and at the end (Day 21 or 42) of the experimental feeding period (Table 2). Flaxseed has the ability to stabilize blood glucose levels by slowing sugar absorption and improving the body’s response to insulin [34,35]. These findings were confirmed in our study, where glucose concentration decreased at the end of the experiment, mainly in the F6 group (Table 2), which is also consistent with the study by Hasimun et al. [36] in diabetic rats and by Klimiuk et al. [37] in fattening pigs with a 2% portion of flaxseed in the feed. Liver transaminases (AST, ALT), ALP, and GGT reflect liver function and the overall health status of the organism [38]. Our results indicate that flaxseed consumption had no adverse effect on liver health, except for a slight elevation of ALT values during the experimental feeding period compared to physiological values [39]. This temporary increase could be caused by stress during the blood sampling procedure [40] or by the action of pro-inflammatory n-6 FAs from flaxseed, antinutrient compounds, increased oxidative stress, or individual sensitivity [41]. In contrast, at the end of the experiment, ALT activity significantly decreased (see Table 2), probably due to the action of polyphenols or n-3 FAs originating from flaxseed, which have the ability to mitigate inflammatory reactions, mainly in the F6 group. Decreased concentrations of AST and ALT were measured in a study by Klimiuk et al. [37] in fattening pigs receiving 2%, 4%, or 6% flaxseed in the feed. Proteins originating from flaxseed could influence protein content in animals [41], which was confirmed in our study in all groups of animals. The most significant elevation was seen in the F6 group, which received 10% flaxseed in the feed for 6 weeks. Dietary supplementation with an appropriate level of flaxseed may support immune function, potentially contributing to improved production performance and the quality and sensory characteristics of animal products [14]. However, the relatively high protein content associated with flaxseed supplementation may increase the metabolic demands placed on the liver and kidneys, potentially contributing to their hypertrophy as an adaptive response to increased protein intake [42].

Lactate dehydrogenase is an important enzyme involved in glycolysis and gluconeogenesis. It is found in almost all body cells and plays a central role in energy metabolism [43]. Similar to our previous study, it can be used as a marker of cell membrane disruption in animals [31]. LDH release into the interstitial space was assessed in blood serum and tissue extracts from the heart, liver, and skeletal muscle of fattening pigs. In blood serum, total LDH and its isoenzymes LDH-2, LDH-3, and LDH-5 were decreased at the end of the feeding period and after 6 weeks (Table 3), which indicates that flaxseed improves cell membrane integrity and reduces leakage of this enzyme (Figure 2). These findings were also confirmed in our previous research [31] in piglets, which received flaxseed in combination with probiotic cheese. Moreover, selected concentrations of isoenzymes supported this theory, e.g., decreased LDH 1 concentration in the heart muscle in the F6 group and decreased LDH 5 activity in the liver and skeletal muscle, whose varying activity is typical for selected organs. Lactate dehydrogenase was also affected by flaxseed in our previous study on mice exposed to xylene [9] and in the study by Alaubody et al. [41].

The liver is the main site for metabolic interactions of PUFAs and other FAs, and their concentration reflects their status in blood serum. Many studies have discussed the effect of flaxseed feeding on PUFA concentrations in blood serum, meat, or other organs in fattening pigs [25,28]. In our study, the highest content of ALA was observed in pigs receiving 10% flaxseed for 6 weeks (Table 4). The high proportion of ALA resulted in a high conversion of n-3 PUFAs at the expense of n-6 PUFAs. Similar statements were reported in our previous research [44] and in the study by Kim et al. [45], Bartkovský et al. [25], and Klimiuk et al. [37] in fattening pigs. In contrast to our study, Smink et al. [46] reported that a high intake of ALA increased EPA but decreased DHA directly in the liver of growing pigs. In our study, both essential FAs were increased at the end of the feeding period. Moreover, the most significant elevation of overall n-3 FAs in the F6 group (24.42 mol%) compared to the control (2.47 mol%) or the F3 group (6.204 mol%) was observed.

A high intake of essential linoleic acid (LA) increased the content of LA and other long-chain n-6 PUFAs such as arachidonic acid (AA) in liver fat. This agrees with other studies in pigs [27,46]. The sum of n-6 FAs was highest in the control animals; therefore, we conclude that 3 weeks of flaxseed application in the pigs’ diet was not sufficient to incorporate n-6 FAs into the liver tissue, as shown in our previous research [25]. Low conversion of n-6 FAs reflects a low concentration of gamma-linolenic acid (GLA) in the F6 group compared to the F3 group, which is a precursor of AA (Table 4). A three-week flaxseed feeding period led to a decreased concentration of AA in the liver of pigs, but this decline was most visible in pigs that received flaxseed for 6 weeks. This reduction is important because overproduction of AA in phospholipid membranes results in overproduction of pro-inflammatory eicosanoids [3]. Better utilization and conversion of n-3 FAs originating from flaxseed were visible in pigs after 6 weeks of flaxseed consumption. We measured a higher content of clupanodonic acid (n-3 DPA) and lower osbondic acid (n-6 DPA) in this group of animals compared to fattening pigs that received flaxseed for 3 weeks.

The ideal ratio of n-6 to n-3 fatty acids in the diet should be less than 5:1, with a ratio between 3:1 and 1:1 often considered optimal. Higher intakes of n-6 (typical of the Western diet) promote inflammation, while sufficient n-3 FAs protect the heart and blood vessels [3,47]. The n-6 to n-3 ratio was significantly affected by the addition of 10% flaxseed to the feed of fattening pigs. After 6 weeks of supplementation, this ratio reached a value of 0.932, which is considered optimal for maintaining health benefits. A 3-week supplementation period was also sufficient to incorporate FAs into membranes and reached a value of 1.188. In our study, we also measured the EPA to AA ratio, which highlighted better conversion of n-3 PUFAs at the expense of n-6 PUFAs, thus indicating an anti-inflammatory and cardioprotective effect [48]. Moreover, we also measured the n-3 index, which reflects the red blood cell concentration of EPA, DHA, and others, and it is also a well-validated, reproducible marker in research, representing long-term nutritional status rather than just recent intake [49]. Both parameters (EPA:AA ratio, n-3 index) were significantly elevated in the group of pigs that received flaxseed for a long-term period of 6 weeks. However, a shorter feeding period (3 weeks) also resulted in an elevation of these parameters compared to the control group.

To our knowledge, there are no studies related to the direct effect of flaxseed feeding on micro-vascularization or cell proliferation in the liver and jejunum of fattening pigs. CD31, also known as the platelet endothelial cell adhesion molecule (PECAM 1), is a transmembrane glycoprotein expressed on endothelial cells. It plays a critical role in enhancing endothelial barrier properties and facilitating leukocyte transmigration during inflammation and angiogenesis [50]. In recent studies on pigs, the CD31 antigen was usually detected as a marker of angiogenesis in the placenta [51] or as evidence of the skin healing process [52]. In our study, we measured CD31 in the liver and jejunum of pigs fed flaxseed for 6 or 3 weeks. Our results indicated that higher CD31 immunoreactivity was seen in the F6 group in both organs, which was evidence of better microvascularization. In future experiments, those findings should be studied in detail and supplemented by, e.g., Factor VIII or MIL11 endothelial markers, or quantified by PCR methods.

This study also focused on changes in PCNA (proliferating cell nuclear antigen) expression in porcine liver and jejunum. We observed a significant increase in PCNA expression in fattening pigs fed flaxseed for 6 weeks (Figure 3 and Figure 4). Generally, flaxseed has the ability to decrease PCNA expression in cancerous or damaged cells, where the lignans and n-3 fatty acids from flaxseed could suppress VEGF-mediated pathological angiogenesis [53,54]. Studies on healthy individuals are lacking; therefore, we provide a deeper look into this issue. However, further studies are needed to explain the direct role of flaxseed components in healthy fattening pigs. Ndou et al. [55] studied the effect of flaxseed on the histomorphology of the jejunum and its microbiome. They concluded that flaxseed increases the height of the villi, crypt depth, and other parameters, which could facilitate nutrient absorption. Literature resources regarding pigs’ liver or jejunum after flaxseed consumption are very limited. Jonecová et al. [56] studied flaxseed alone or in combination with probiotics and its effects on histological changes in the jejunum of weaning piglets. The authors claimed that flaxseed alone increased the proliferation rate after 21 days post-weaning in those piglets and that synbiotics decreased proliferation.

Future studies should include a larger number of animals. Another limitation is that we should precisely record the exact amount of food consumed by animals and food debris to calculate average daily feed intake (ADFI), feed to gain ratio (F/G ratio), and other production performance parameters. In subsequent studies, it is suitable to measure fatty acid composition in other tissues, such as muscles, adipose tissue, the aorta, the brain, and others, to support our hypothesis about the beneficial effect of flaxseed consumed at a 10% concentration for 6 weeks. In future experiments with healthy fattening pigs, a direct effect of flaxseed on cell membrane integrity should be supported by detailed histological and immunohistochemical analysis, such as markers of cell apoptosis, inflammation, antioxidant status, and especially tight junction proteins. Some of them could be detected by PCR methods and supported by testing the hypothesis on cell cultures.

## 5. Conclusions

Our study highlighted the beneficial effect of flaxseed feeding in fattening pigs. Especially, a six-week supplementation period seems to be suitable for maintaining animal health by lowering blood glucose levels, balancing basic biochemical enzymes, and reducing the leakage of LDH, thus improving cell membrane integrity. Moreover, the long-term feeding period led to better utilization of essential fatty acids, reflected in the elevation of ALA, EPA, DHA, and AA compared to the short-term feeding period. ALA originating from flaxseed inhibits the conversion of LA into n-6 PUFAs and thus improves the n-6:n-3 ratio and lowers other atherogenic indices. Results of the protein expressions of CD31 and PCNA suggest that a 6-week supplementation period is more suitable for improving cell proliferation or initiating repair processes and micro-vascularization of the liver and villi, which lead to better nutrient absorption and promote gut health. The direct effect of flaxseed needs to be elucidated in future experiments.

## Figures and Tables

**Table 1:** Nutritional composition of the standard feed mixture, flaxseed and their combination, expressed on a 100% dry matter basis, and fatty acid composition of the respective feed types expressed as mol%.

Items	SFM	F	SFM + 10% F	Fatty Acid	SFM	SFM + 10% F
Crude protein, g/kg	140.42	227.6	150.51	14:0 (mol%)	0.101	0.050
Crude fat, g/kg	18.7	307.25	42.23	16:0 (mol%)	14.281	4.496
Crude fiber, g/kg	35.69	232.58	68.29	18:0 (mol%)	1.899	2.547
Neutral detergent fiber, g/kg	176.11	411.19	189.04	18:2n-6 (mol%)	55.73	8.547
Acid detergent fiber, g/kg	42.27	278.9	74.47	18:3n-6 (mol%)	0.09	0.018
Ash, g/kg	60.12	35.5	48.30	18:3n-3 (mol%)	7.109	72.546
Starch, g/kg	574.62	42.32	510.28	20:4n-6 (mol%)	0.691	0.052
Ca, g/kg	8.12	2.81	12.80	20:5n-3 (mol%)	0.125	0.0003
Mg, g/kg	3.35	4.11	3.59	22:5n-6 (mol%)	0.245	0.022
Na, g/kg	1.45	4.33	1.52	22:5n-3 (mol%)	0.043	0.140
K, g/kg	5.58	8.12	6.29	22:6n-3 (mol%)	0.073	0.055
P, g/kg	4.80	2.71	5.84	Ʃ n-3	7.349	72.741
Cu, mg/kg	40.82	33.55	57.40	Ʃ n-6	57.227	8.720
Zn, mg/kg	131.83	48.7	103.45	n-6:n-3 ratio	7.787	0.120
Mn, mg/kg	150.57	43.83	125.80	EPA:AA ratio	0.181	0.005
Metabolizable energy, MJ/kg	13.26	12.87	13.36	n-3 index	7.349	72.741

SFM, standard feed mixture; F, flaxseed; n-3 index, sum of 18:3n-3 + 20:5n-3 + 22:5n-3 + 22:6n-3 acids; the concentration of fatty acids is expressed as a percentage of total fatty acids.

**Table 2:** Mean values of selected blood parameters in fattening pigs.

Parameter	Groups	Blood Collection		
		First	Second	Third
Glucose (mmol/L)	C	5.2 ± 0.18	5.27 ± 0.89	5.45 ± 0.29
	F6	4.92 ± 0.41	4.43 ± 0.13	4.6 ± 0.21
	F3	4.91 ± 0.4	4.8 ± 0.38	5.18 ± 0.11
AST (µkat/L)	C	1.26 ± 0.3	1.28 ± 0.25	1.29 ± 0.25
	F6	0.96 ± 0.09	0.74 ± 0.06	0.98 ± 0.05
	F3	0.96 ± 0.09	0.78 ± 0.08	1.02 ± 0.04
ALT (µkat/L)	C	1.27 ± 0.05	1.15 ± 0.07	1.14 ± 0.09
	F6	1.12 ± 0.04	0.87 ± 0.07	1.05 ± 0.06
	F3	1.12 ± 0.04	0.99 ± 0.06	1.00 ± 0.02
ALP (µkat/L)	C	5.26 ± 0.3	3.45 ± 0.36	4.42 ± 0.43
	F6	4.89 ± 0.44	3.38 ± 0.6	3.58 ± 0.55
	F3	4.89 ± 0.44	4.07 ± 0.16	4.84 ± 0.27
GGT (µkat/L)	C	0.66 ± 0.05	0.51 ± 0.05	0.49 ± 0.03
	F6	0.55 ± 0.06	0.52 ± 0.1	0.77 ± 0.09
	F3	0.55 ± 0.06	0.52 ± 0.07	0.84 ± 0.13
CK (µkat/L)	C	38.53 ± 14.66	35.11 ± 15.07	39.02 ± 8.22
	F6	13.51 ± 2.27	29.03 ± 6.31	23.65 ± 3.98
	F3	13.51 ± 2.27	50.62 ± 15.24	31.50 ± 3.65
TP (g/L)	C	62.93 ± 1.67	66.70 ± 1.93	67.17 ± 1.43
	F6	63.05 ± 1.29	68.22 ± 2.16	72.73 ± 1.94
	F3	63.05 ± 1.29	66.55 ± 1.01	68.00 ± 1.26

C, control group; F6, flaxseed fed for 6 weeks; F3, flaxseed fed for 3 weeks; First, first blood sampling or Day 0 of the experiment; Second, second blood sampling (Day 11 or 21); Third, third blood sampling or the end of the experiment (Day 21 or 42); AST, aspartate aminotransferase; ALT, alanine aminotransferase; GGT, gamma-glutamyl transferase; CK, creatine kinase; ALP, alkaline phosphatase; TP, total protein. Values in Table 2 are presented as mean ± SEM.

**Table 3:** Table presents within-group pairwise comparisons between blood collection time points and between-group pairwise comparisons at each blood collection time point in selected blood parameters of fattening pigs.

Parameter	Within-Group Pairwise Comparisons Between Blood Collection Time Points				Parameter	Between-Group Pairwise Comparisons at Each Blood Collection Time Point			
	Group	First vs. Second	First vs. Third	Second vs. Third		Blood Collection	C vs. F6	C vs. F3	F6 vs. F3
Glucose	C	ns	ns	ns	Glucose	First	ns	ns	ns
	F6	ns	ns	ns		Second	ns	ns	ns
	F3	ns	ns	ns		Third	*	ns	ns
AST	C	ns	ns	ns	AST	First	ns	ns	ns
	F6	ns	ns	ns		Second	*	ns	ns
	F3	ns	ns	ns		Third	ns	ns	ns
ALT	C	***	***	ns	ALT	First	ns	ns	ns
	F6	**	ns	*		Second	*	ns	ns
	F3	ns	ns	ns		Third	ns	ns	ns
ALP	C	***	ns	***	ALP	First	ns	ns	ns
	F6	*	ns	ns		Second	ns	ns	ns
	F3	ns	ns	ns		Third	ns	ns	ns
GGT	C	***	***	ns	GGT	First	ns	ns	ns
	F6	ns	ns	ns		Second	ns	ns	ns
	F3	ns	ns	ns		Third	*	*	ns
CK	C	ns	ns	ns	CK	First	ns	ns	ns
	F6	ns	ns	ns		Second	ns	ns	ns
	F3	***	ns	ns		Third	ns	ns	*
TP	C	ns	ns	ns	TP	First	ns	ns	ns
	F6	ns	***	ns		Second	ns	ns	ns
	F3	ns	ns	ns		Third	*	ns	ns

Statistical significance in Table 3 is indicated as follows: ns, *p* ≥ 0.05; *, *p* < 0.05; **, *p* < 0.01; ***, *p* < 0.001.

**Table 4:** Lactate dehydrogenase activity in blood serum of fattening pigs during the flaxseed supplementation period.

Parameter (µkat/L)	Groups	Blood Collection		
		First	Second	Third
TLDH	C	16.1 ± 2.25	14.8 ± 2.43	19.85 ± 1.95
	F6	12.37 ± 0.4	16.85 ± 0.98	15.23 ± 0.61
	F3	12.37 ± 0.4	11.88 ± 1	16.85 ± 0.98
LDH-1	C	4.58 ± 0.17	7.42 ± 1	7.01 ± 0.54
	F6	6.4 ± 0.12	4.55 ± 0.35	7.06 ± 0.29
	F3	5.4 ± 0.14	5.3 ± 0.13	6.9 ± 0.21
LDH-2	C	1.33 ± 0.04	1.43 ± 0.1	1.65 ± 0.05
	F6	1.39 ± 0.12	1.29 ± 0.07	0.94 ± 0.04
	F3	1.44 ± 0.14	1.37 ± 0.1	1.32 ± 0.11
LDH-3	C	1 ± 0.11	1.31 ± 0.25	1.46 ± 0.06
	F6	1.44 ± 0.12	1.06 ± 0.06	0.91 ± 0.11
	F3	1.38 ± 0.07	1.41 ± 0.19	1.89 ± 0.23
LDH-4	C	1.32 ± 0.16	1.86 ± 0.36	1.92 ± 0.07
	F6	2.06 ± 0.14	1.27 ± 0.04	1.88 ± 0.07
	F3	1.89 ± 0.19	2.03 ± 0.13	2.51 ± 0.26
LDH-5	C	4.26 ± 1.33	2.04 ± 0.35	4.79 ± 0.37
	F6	3.92 ± 0.49	3.59 ± 0.57	1.64 ± 0.22
	F3	2.26 ± 0.09	1.77 ± 0.22	4.23 ± 0.33

C, control group; F6, flaxseed fed for 6 weeks; F3, flaxseed fed for 3 weeks; First, first blood sampling or Day 0 of the experiment; Second, second blood sampling (Day 11 or 21); Third, third blood sampling or the end of the experiment (Day 21 or 42); TLDH, total lactate dehydrogenase; LDH-1–LDH-5, lactate dehydrogenase isoenzymes 1–5. Values in Table 4 are presented as mean ± SEM.

**Table 5:** The table presents within-group pairwise comparisons between blood collection time points and between-group pairwise comparisons at each blood collection time point in serum lactate dehydrogenase activity.

Parameter	Within-Group Pairwise Comparisons Between Blood Collection Time Points				Parameter	Between-Group Pairwise Comparisons at Each Blood Collection Time Point			
	Group	First vs. Second	First vs. Third	Second vs. Third		Blood Collection	C vs. F6	C vs. F3	F6 vs. F3
TLDH	C	ns	ns	*	TLDH	First	ns	ns	ns
	F6	ns	ns	ns		Second	ns	ns	ns
	F3	**	**	**		Third	*	ns	ns
LDH-1	C	*	*	*	LDH-1	First	ns	ns	ns
	F6	***	***	***		Second	*	ns	ns
	F3	ns	ns	ns		Third	ns	ns	ns
LDH-2	C	ns	*	ns	LDH-2	First	ns	ns	ns
	F6	ns	*	ns		Second	ns	ns	ns
	F3	ns	ns	ns		Third	***	ns	ns
LDH-3	C	ns	***	ns	LDH-3	First	ns	ns	ns
	F6	ns	**	ns		Second	ns	ns	ns
	F3	ns	ns	ns		Third	**	ns	ns
LDH-4	C	ns	ns	ns	LDH-4	First	***	ns	ns
	F6	***	ns	**		Second	ns	ns	ns
	F3	ns	***	ns		Third	ns	ns	ns
LDH-5	C	ns	ns	**	LDH-5	First	ns	ns	ns
	F6	ns	**	*		Second	ns	ns	ns
	F3	ns	**	ns		Third	***	ns	***

Statistical significance in Table 5 is indicated as follows: ns, *p* ≥ 0.05; *, *p* < 0.05; **, *p* < 0.01; ***, *p* < 0.001. Table 5 presents within-group pairwise comparisons between blood collection time points and between-group pairwise comparisons at each blood collection time point.

**Table 6:** Liver fatty acid content in fattening pigs.

Fatty Acids	Trivial Name (Abbreviation)	C	F6	F3
12:0 (mol%)	Lauric	0.048 ± 0.008	0.095 ± 0.059	0.128 ± 0.033
14:0 (mol%)	Myristic	0.968 ± 0.119	0.964 ± 0.559	1.519 ± 0.354
16:0 (mol%)	Palmitic	24.24 ± 1.357	23.82 ± 2.36	26.88 ± 1.653
18:0 (mol%)	Stearic	6.631 ± 1.099	15.46 ± 3.412	2.759 ± 0.285
18:2n-6 (mol%)	Linoliec (LA)	30.17 ± 0.524	13.44 ± 0.537	15.39 ± 0.74
18:3n-6 (mol%)	Gamma-linolenic (GLA)	0.091 ± 0.018	0.131 ± 0.035	0.515 ± 0.061
18:3n-3 (mol%)	Alfa-linolenic (ALA)	1.432 ± 0.076	5.952 ± 0.518	1.936 ± 0.264
20:3n-6 (mol%)	Dihomo-gamma-linoleic (DGLA)	0.419 ± 0.166	1.832 ± 0.377	0.5919 ± 0.113
20:4n-6 (mol%)	Arachidonic (AA)	5.293 ± 0.903	1.913 ± 0.192	2.155 ± 0.258
20:5n-3 (mol%)	Timnodonic (EPA)	0.056 ± 0.002	12.31 ± 0.486	2.501 ± 0.415
22:5n-6 (mol%)	Osbond (DPA-6)	0.12 ± 0.035	0.114 ± 0.011	0.484 ± 0.041
22:5n-3 (mol%)	Clupanodonic (DPA-3)	0.229 ± 0.091	1.539 ± 0.26	0.353 ± 0.046
22:6n-3 (mol%)	Cervonic (DHA)	0.754 ± 0.307	4.619 ± 0.251	1.414 ± 0.203
Ʃ n-3		2.47 ± 0.34	24.42 ± 0.89	6.204 ± 0.535
Ʃ n-6		37.48 ± 1.73	22.59 ± 0.264	21.55 ± 1.428
n-6:n-3 ratio		16.09 ± 1.483	0.932 ± 0.038	3.537 ± 0.242
EPA:AA ratio		0.014 ± 0.004	6.787 ± 0.703	1.188 ± 0.148
n-3 index		2.47 ± 0.34	24.42 ± 0.89	6.204 ± 0.535

C, control group; F6, flaxseed fed for 6 weeks; F3, flaxseed fed for 3 weeks; LA, linoleic acid; GLA, gamma-linolenic acid; ALA, alpha-linolenic acid; DGLA, dihomo-gamma-linolenic acid; AA, arachidonic acid; EPA, timnodonic acid; DPA-6, osbond acid; DPA-3, clupanodonic acid; DHA, cervonic acid. The n-3 index represents the sum of 18:3n-3, 20:5n-3, 22:5n-3, and 22:6n-3 fatty acids. Fatty acid concentrations are expressed as a percentage of total fatty acids. Values in Table 6 are presented as mean ± SEM.

**Table 7:** Between-group pairwise comparisons in liver fatty acid content of fattening pigs.

Fatty Acid/Index	C vs. F6	C vs. F3	F6 vs. F3
12:0 Lauric	ns	*	ns
14:0 Myristic	ns	ns	ns
16:0 Palmitic	ns	ns	ns
18:0 Stearic	*	**	**
18:2n-6 LA	***	***	ns
18:3n-6 GLA	ns	***	***
18:3n-3 ALA	***	ns	***
20:3n-6 DGLA	***	ns	*
20:4n-6 AA	**	**	ns
20:5n-3 EPA	***	ns	***
22:5n-6 DPA-6	ns	***	***
22:5n-3 DPA-3	***	ns	**
22:6n-3 DHA	***	ns	***
Σ n-3	***	ns	***
Σ n-6	***	***	ns
n-6:n-3 ratio	***	***	***
EPA:AA ratio	***	ns	***
n-3 index	***	ns	***

Statistical significance of between-group pairwise comparisons in Table 7 is indicated as follows: ns, *p* ≥ 0.05; *, *p* < 0.05; **, *p* < 0.01; ***, *p* < 0.001.

## Data Availability

All the data appears in the manuscript. For further inquiries, please contact the corresponding author.

## Abbreviations

The following abbreviations are used in this manuscript:

C	Control group
F6	Flaxseed Fed for 6 Weeks
F3	Flaxseed Fed for 3 Weeks
FA	Fatty Acid
PUFA	Polyunsaturated Fatty Acid
ALA	Alfa-linolenic Acid
LA	Linoliec Acid
AA	Arachidonic Acid
EPA	Timnodonic Acid
DHA	Cervonic Acid
GLA	Gamma-linolenic Acid
DGLA	Dihomo-gamma-linoleic Acid
DPA-3	Clupanodonic Acid
DPA-6	Osbond Acid
ROD	Relative Optical Density
LDH	Lactate Dehydrogenase
ALT	Alanine Aminotransferase
AST	Aspartate Aminotransferase
GGT	Gamma-glutamyl Transferase
CK	Creatine Kinase
ALP	Alkaline Phosphatase
TP	Total Protein
CD31	Platelet Endothelial Cell Adhesion Molecule
PCNA	Proliferating Cell Nuclear Antigen
SEM	Standard Error of the Mean
